# Distinct metabolite classes in root exudates are indicative for field- or hydroponically-grown cover crops

**DOI:** 10.3389/fpls.2023.1122285

**Published:** 2023-04-06

**Authors:** Diana Heuermann, Stefanie Döll, Dörte Schweneker, Ulf Feuerstein, Norman Gentsch, Nicolaus von Wirén

**Affiliations:** ^1^ Physiology and Cell Biology, Leibniz Institute of Plant Genetics and Crop Plant Research Gatersleben, Seeland, Germany; ^2^ Stress and Developmental Biology, Leibniz Institute of Plant Biochemistry, Halle (Saale), Germany; ^3^ Deutsche Saatveredelung Aktiengesellschaft (AG), Asendorf, Germany; ^4^ Institute of Soil Science, Leibniz Universität Hannover, Hannover, Germany

**Keywords:** Root exudates, untargeted metabolite profile, primary metabolites, secondary metabolites, root washing, hydroponics, cover crops

## Abstract

**Introduction:**

Plants release a large variety of metabolites via their roots to shape physico-chemical soil properties and biological processes in the rhizosphere. While hydroponic growth conditions facilitate accessibility of the root system and recovery of root exudates, the natural soil environment can alter root metabolism and exudate secretion, raising the question to what extent the quantity and composition of root exudates released in hydroponic growth systems reflect those recovered from soil-grown roots.

**Methods:**

Using a root washing method, we sampled root exudates from four field-grown cover crop species with wide taxonomic distance, namely white mustard, lacy phacelia, bristle oat, and Egyptian clover. A set of primary metabolites and secondary metabolites were analysed in a targeted and untargeted LC-MS-based approach, respectively, for comparison with exudates obtained from hydroponically cultured plants.

**Results and discussion:**

We found that hydroponically cultivated plants released a larger amount of total carbon, but that the recovery of total carbon was not indicative for the diversity of metabolites in root exudates. In the field, root exudates from phacelia and clover contained 2.4 to 3.8 times more secondary metabolites, whereas carbon exudation in hydroponics was 5- to 4-fold higher. The composition of the set of metabolites identified using the untargeted approach was much more distinct among all species and growth conditions than that of quantified primary metabolites. Among secondary metabolite classes, the presence of lipids and lipid-like molecules was highly indicative for field samples, while the release of a large amount of phenylpropanoids, organoheterocyclic compounds or benzenoids was characteristic for clover, mustard or oat, respectively, irrespective of the cultivation condition. However, at the compound level the bulk of released metabolites was specific for cultivation conditions in every species, which implies that hydroponically sampled root exudates poorly reflect the metabolic complexity of root exudates recovered from field-grown plants.

## 1 Introduction

Plants release chemical compounds *via* their roots and thereby affect chemical and biological processes in the root-surrounding soil, the so-called rhizosphere (Hinsinger et al., 2005). These root exudates are released to mobilize sparingly available nutrients, to communicate with other plants or soil organisms, e.g. to shape a beneficial rhizosphere microbial community, to defend themselves against soil pests and pathogens, to detoxify metals outside the root (Herz et al., 2018; Preece and Peñuelas, 2020), or to protect roots from desiccation during phases of low soil moisture content (Williams and Vries, 2019). To acquire phosphorus (P), for instance, acid phosphatases are secreted that mobilize P from organic sources (Sun et al., 2020), while inorganic phosphate can be released from precipitates with iron (Fe), aluminum or calcium by proton release and subsequent rhizosphere acidification or when those cations become chelated by secreted organic acids or phenolic compounds (Dakora and Phillips, 2002; Sun et al., 2020). Besides those root exudates with less nutrient-specific mobilizing properties, there are others with specific modes of action. Under suboptimal Fe supply, roots of graminaceous plant species secrete mugineic acid-type phytosiderophores to chelate Fe^3+^ in the rhizosphere for subsequent uptake of intact Fe^3+^-phytosiderophore complexes (Schaaf et al., 2004; Chen et al., 2017), while most dicot species release catecholic coumarins that can chelate and reduce Fe(III) for delivery and subsequent uptake of ionic Fe^2+^ (Schmid et al., 2014; Rajniak et al., 2018).

Besides mobilizing nutrients, root-released exudates can also shape the root microbiome and attract soil microbes beneficial for nutrient acquisition. Secreted sugars and strigolactones attract mycorrhizal fungi and promote colonization with those symbiotic partners that are most efficient in P mobilization (Crombez et al., 2019). To improve nitrogen (N) acquisition, different crop species release flavonoids to attract symbiotic or associative rhizobacteria allowing them to benefit from their atmospheric N_2_-fixation or their production of growth-stimulating compounds (Roy et al., 2019; Yu et al., 2021). Defense against soil pathogens is mediated by chemical compounds mostly of low molecular weight, like amino acids, organic acids, sugars, phenols or terpenoids. Those compounds are released constitutively as phytoanticipins or can be induced by specific pathogens, then called phytoalexins (Baetz and Martinoia, 2014). In addition, root exudates can attract beneficial bacteria that suppress pathogen populations in the soil (Olanrewaju et al., 2017). Root exudates mediate communication also with other plants in the immediate surrounding (Kong et al., 2018). Apart from volatile compounds, plants can recognize the presence of neighbors by their root exudate pattern irrespective of whether those neighbors are closely related or from an unrelated plant species (van Dam and Bouwmeester, 2016). Major allelopathic compounds which are released in order to compete against neighbors belong to the classes of phenols, coumarins, lignans, flavonoids, tannins, benzoxazolinones, glucosinolates, amino acids, alkaloids, polyacetylenes, terpenes or apocarotinoids and can impair seed germination, seedling growth, root elongation, biomass accumulation of roots and shoots or nutrient uptake of neighbors (Flamini, 2012).

The release of root exudates to influence rhizosphere processes and interactions with their biotic and abiotic surrounding represents always a trade-off between advantages for the plant and energy loss (Preece and Peñuelas, 2020). In a meta study, Pausch and Kuzyakov (2018) calculated that crops invest on average 7% of their photosynthetically fixed carbon (C) into rhizodeposit. In less-intensively bred grasses even 11% of totally fixed C were allocated to the rhizosphere. To a certain extent this superior exudation rate appears related to different root exudate patterns, in a way that wild relatives of crops can be more effective in nutrient acquisition, establishment of symbiotic interactions and defense against pathogens than modern crop species (Preece and Peñuelas, 2020). So far, only a few root exudate components have been identified and placed into ecological context, while the large majority has remained little or un-characterized.

Cover crops are employed to protect or even improve soil quality and included in crop rotations during periods with unfavorable growth conditions for cash crops (Dabney et al., 2001). Their cultivation is beneficial for the reduction of nutrient losses through nutrient fixation in their biomass and for improving nutrient availability for the following crop, as well as for lowering soil erosion and improving soil quality (Gentsch et al., 2020). Furthermore, cover crops can be effective in controlling soil-borne pests and pathogens (Hossain et al., 2012; Mielniczuk et al., 2020) or the establishment of weeds (Kwiatkowski et al., 2016). Therefore, cover crop cultivation is a highly valuable management practice to reduce fertilizer and pesticide inputs in agricultural plant production (Preece and Peñuelas, 2020). In part, the beneficial properties of cover crops have been linked to the action of root exudates (Pérez and Ormeño-Nuñez, 1991; Sherif et al., 2013; Li et al., 2019; Hazrati et al., 2021). With regard to their less intense breeding history (Wayman et al., 2017), cover crop species might bear a large potential for the discovery and improvement of beneficial root exudate-related traits.

Hydroponic growth conditions allow easy accessibility of the root system and are thus often used in root exudate sampling approaches (Oburger and Jones, 2018). However, this cultivation method is far away from a natural soil environment where roots have physical contact with soil particles, interfere with soil organisms or require more energy to acquire nutrients from sparingly-available sources (van Dam and Bouwmeester, 2016; Oburger and Jones, 2018). Analyzing root exudates from soil-grown plants will thus yield much more realistic metabolite profiles (Herz et al., 2018). However, a soil matrix comes with several difficulties, in particular by metabolite adsorption to soil particles (van Dam and Bouwmeester, 2016) and microbial metabolite transformation or degradation (Oburger and Jones, 2018). Such problems can be partly circumvented by washing roots before exudate sampling in water as shown e.g., by Herz et al. (2018) and Dietz et al. (2019), but this method bears the risk of damaging roots which can lead to a change in the metabolite profile of root exudates (Oburger and Jones, 2018). In this context, an open question is to what extent the quantity and composition of root exudates released in hydroponic growth systems reflect those recovered from soil-grown roots.

To address the question of comparability between soil-grown and hydroponic root exudates, we used a root washing method to sample root exudates from four field-grown cover crop species, namely white mustard (*Sinapis alba* L.), lacy phacelia (*Phacelia tanacetifolia* Benth.), bristle oat (*Avena strigosa* Schreb.) and Egyptian clover (*Trifolium alexandrinum* L.), and compared targeted profiles of a set of 28 primary metabolites and LC-MS-based untargeted metabolite profiles with exudates obtained from hydroponically cultured plants. Due to the wide taxonomic distance among these species, we expected to end up with highly specific root-exuded metabolite patterns (Reinhold-Hurek et al., 2015), and asked how much the hydroponic exudate profiles can tell about those of field-grown plants. We hypothesized (i) that the exudation rate of total C is positively related to the number of released and detected metabolic compounds, (ii) that the pattern of secondary metabolites is more characteristic for plant species as well as for cultivation conditions than that of primary metabolites, and (iii) that certain metabolites and metabolite classes are indicative for root exudates of individual species and cultivation conditions.

## 2 Materials and methods

### 2.1 Plant material and growth conditions

#### 2.1.1 Plant material

Four cover crop species were used in this study: White mustard (*Sinapis alba*) cv. Litember, lacy phacelia (*Phacelia tanacetifolia*) cv. Bee Happy, bristle oat (*Avena strigosa*) cv. Panache and Egyptian clover (*Trifolium alexandrinum*) cv. Alex. Seeds were obtained from the Deutsche Saatveredelung, Lippstadt, Germany.

#### 2.1.2 Hydroponic cultivation

Seeds of the four cover crop species were pre-cultured for 7 days using the sandwich method (Koeslin-Findeklee et al., 2015). Thereafter, seedlings were rolled into sponges and mounted onto 5 L hydroponic growth vessels and placed in continuously randomized positions in a phytochamber. The growth vessels were equipped with pipes for aeration of the nutrient solution, establishing an oxygen concentration of ~7.6 mg/l (measured with Microx 4 oxygen sensor, PreSense, Regensburg, Germany). Four plants per cover crop species shared one vessel. In total, 18-21 pots have been installed per species. The nutrient solution contained 1 mM NH_4_NO_3_, 1 mM Ca(NO_3_)_2_, 0.75 mM MgSO_4_, 0.75 mM KH_2_PO_4_, 0.4 mM K_2_SO_4_, 0.3 mM Fe-EDTA, 0.2 mM CaCl_2_, 0.02 mM H_3_BO_3_, 4 µM MnSO_4_, 0.75 µM ZnSO_4_, 0.6 µM CuSO_4_ and 0.2 µM NaMoO_4_; pH was adjusted to 5.8 using KOH. The solution was changed twice a week until day 16, afterwards it was replaced daily. Each time, all pots were randomly shuffled to another position in the phytochamber after changing the nutrient solution to avoid position effects. The light phase was set at 300 µmol s^-1^ m^-2^ and 25°C for 16 hours. In the 8-hour dark phase, temperature was 20°C. Air humidity was at 60% during the whole day.

#### 2.1.3 Field cultivation

The four cover crop species were grown on an experimental site in Asendorf, Lower-Saxony, Germany. This field site is located 49 m above sea level at 52°45′48.4″N 9°01′24.3″E and has a mean annual temperature of 9.3°C and mean annual precipitation of 751 mm. The soil developed from shallow loess over glaciofluvial sand (>50 cm) and was classified as Stagnic-Cambisol (IUSS Working Group, 2015). Basic soil characteristics of different soil depths are given in Gentsch et al. (2020). Soil texture was silty loam (20% sand, 73% silt, 7% clay) and soil pH was 6.0 in the topsoil and 6.4 in the subsoil. Soil organic carbon content decreased from 1.73% in 0-10 cm to 0.97% in 30-60 cm soil depth.

On 16.8.2018, cover crop stands were established in a randomized complete block design including plots of 9 x 9 m in three replicates. Winter wheat (*Triticum aestivum* L.) cv. Patras was the preceding crop on the field site, whose straw was chopped one month before cover crop sowing and incorporated 12 cm deep into the soil with a cultivator on 13.8.2018. On 28.8.2018, 47 kg N ha^-1^ were given in the form of urea ammonium nitrate solution (UAN) 28. In order to obtain comparable shoot biomasses, seeding strengths varied among cover crops due to differences in the juvenile development of the species (mustard: 300 seeds m^-2^, phacelia: 706 seeds m^-2^, oat: 588 seeds m^-2^, clover: 833 seeds m^-2^).

On 6.9.2018, two holes with a width of 20 x 20 cm and a depth of 30 cm were dug in each cover crop plot. Eight cover crop seedlings were carefully taken from each excavated soil sample and kept on water. Then, the soil was sieved through a 0.5 cm sifter to remove straw rests and filled back into the holes, which were lined with 20 µm polyamide meshes (Franz Eckert GmbH, Waldkirch, Germany). Inside each mesh, eight cover crop seedlings were planted and watered with 3 L of tap water. After one week, the four poorest-performing seedlings were removed from the meshes. Growing plants inside 20 µm-meshes aimed at mimicking an active cover crop stand and allowing microbial- and metabolite-based exchange between roots as well as above-ground competition for light, but offering the opportunity for withdrawing plants with their whole root system since roots were not able to grow out of the meshes.

Weather conditions during the cover crop cultivation in 2018 were as follows [average temperature/precipitation sum]: August – 19.6°C/23.4 mm, September – 15.5°C/41.0 mm, October – 11.3°C/41.0 mm. Due to the extreme drought during that season, cover crops were watered with 40 and 15 mm in August and September, respectively, using an irrigation system (IRTEC, Castelvetro di Modena, Italy).

### 2.2 Sampling and concentration of root exudates

#### 2.2.1 Root exudate sampling in hydroponics

After 29 days of hydroponic cultivation, root exudates from cover crops were sampled. At that time point, developmental stages of hydroponically-grown plants were comparable to field-grown plants in mid-October to avoid the error of development-related differences in the metabolite profile of root exudates. Mustard plants were already flowering (BBCH stage 60-65), while phacelia, oat and clover plants were in the stem elongation phase corresponding to BBCH35-39 (Lancashire et al., 1991). Two hours after the beginning of the light phase (Carvalhais et al., 2011), roots of all four plants from one hydroponic growth vessel were ducked three times in deionized water and placed together in darkened 4.5 L vessels containing deionized water. Root exudates were sampled for four hours at continuous aeration and then frozen at -80°C. All roots from one hydroponic vessel were merged and freeze-dried (lyophilizer from Christ, Osterode, Germany). Deionized water, which was kept for the whole sampling period in hydroponic growth vessels without plants served as “water blank” for controlling background effects in subsequent metabolite analysis. Exudates and blanks were freeze-dried in several portions in 1 L plastic containers (Baumann Saatzuchtbedarf, Waldenburg, Germany). Per container, 10 mL of 50% Ultra Performance Liquid Chromatography (UPLC)-grade methanol were used to resolve exudates on a lab shaker at 4°C overnight. All resolved exudate portions belonging to one sample were merged and concentrated in a vacuum centrifuge (Christ, Osterode, Germany). Then, samples were resolved in 0.75 µl deionized water per mg root dry weight for analyses of primary metabolites. A portion of the remaining sample was concentrated again in a vacuum centrifuge (Christ, Osterode, Germany) and resolved in 0.25 µl 50% methanol per mg root dry weight for non-targeted metabolite analyzes.

#### 2.2.2 Root exudate sampling in the field

After ~5 weeks of cultivation, the plants in the field were at comparable development stages as in the hydroponic system. Only clover was in a slightly earlier phase of shoot development, namely at BBCH33-35, compared to hydroponic system (Lancashire et al., 1991). We aspired to start exudate sampling in the field in a time interval after sunrise which is close to the two hours after beginning of the light phase that were set in hydroponics. Since root washing was very laborious in pre-tests, each replicated field plot per species was sampled on a separate day, namely on days 34, 35 or 36 of cultivation. In early morning, meshes were pulled out of the soil and roots were washed out carefully in boxes with tap water. After washing, roots were kept on tap water until washing of all samples had been finished. This washing procedure posed the risk of damaging cells so that intracellular metabolites, which are usually not released by plant roots, may contaminate the root exudate sampling solution (Oburger and Jones, 2018). In order to remove those metabolites roots were ducked three times in deionized water. Additionally, we checked for the potential amount of cell damage in pre-tests by staining individual roots from all four species after washing with 0.4% trypan blue solution for 1 min (modified from Díaz-Tielas et al. (2012)) and found only few lesions in oat, clover and phacelia (VHX-5000 Digital Microscope, Keyence, Neu-Isenburg, Germany). Only mustard showed blue staining pointing to cell damages that increase the risk for contamination of root exudate samples by intracellular metabolites (**Figure S1**).

Where separation of roots was possible, two plants per species were placed in plastic bottles which were darkened with aluminum foil, filled with 1 L of deionized water and were continuously aerated. Otherwise, all four plants originating from one mesh were placed onto the sampling solution. In total, we took 9-11 biological replicates of root exudates from each species. Due to the higher microbial burden of roots washed from field soil compared to hydroponically-grown roots, microbial activity and metabolic conversion of root exudates during sampling was suppressed by addition of 5 mg L^-1^ Micropur^®^ forte (Katadyn, Mörfelden-Walldorf, Germany) to the deionized water (Oburger et al., 2014). Sampling of root exudates was undertaken for only two hours to avoid re-uptake of root exudates from the lower sampling volume of only 1 L in the field compared to 4.5 L in hydroponics at similar plant density per sampling vessel. Bottles with sampling solution but without plants served as “water blanks” (Dietz et al., 2019). Plants were carefully removed, root fresh weights were determined and sampling solutions were frozen on dry ice. Plant material was dried at 80°C until constant weight and dry mass of the plant parts was determined. Root exudate solution was stored at -80°C until further use. Freeze-drying and resuspension of root exudates was done as described for hydroponics, while exudates were concentrated in 0.75 µl 50% methanol per mg root dry weight in order to obtain sufficient sample volume for metabolite analyses.

### 2.3 Determination of total carbon and metabolite analyzes in root exudates

#### 2.3.1 Total carbon

Total C was determined in 20 µl of root exudate samples, lyophilized in tin capsules, with an elemental analyzer (EuroEA3000, Hekatech, Wegberg, Germany). C concentration was quantified based on a 2.5-bis(5tert-butyl-2-benzo-oxazol-2-yl)thiophen (BBOT) standard using the Callidus software (Hekatech, Wegberg, Germany).

#### 2.3.2 Amino acids

AAs were derivatized with the fluorescent agent aminoquinolyl-N-hydroxysuccimidyl carbamate (AQC), which reacts with the amino group to form stable and strongly fluorescing urea-like structures (after Cohen and Michaud, 1993). 3 mg AQC, prepared at IPK Gatersleben, were dissolved in 1 ml pure acetonitrile. For derivatization, 160 µl 0.2 M boric acid pH 8.8 and 20 µl root exudate were mixed with 20 µl AQC reagent and incubated for 10 min at 55°C and then centrifuged for 1 min at 14000 rpm.

The AQC-derivatized AAs were separated using UPLC (Acquity H-Class system, Waters, Eschborn, Germany) with a C18 reversed-phase column (AccQ-Tag Ultra C18, 1.7 µm, 2.1x100 mm, Waters, Eschborn, Germany) and four different eluents: Eluent A (pure “Eluent A Concentrate” from Waters, Eschborn, Germany), eluent B (90% LC-MS grade water + 10% “Eluent B Concentrate” from Waters, Eschborn. Germany), eluent C (pure “Eluent B Concentrate”) and eluent D (pure LC-MS grade water). The column was equilibrated with eluent A (10%) and eluent C (90%) for at least 30 min. The gradient for sample run was produced as follow: 0 min 10% A and 90%C/0.29 min 9.9% A and 90.1% C/5.49 min 9% A, 80% B and 11% C/7.1 min 8% A, 15.6% B, 57.9% C and 18.5% D/7.3 min 8% A, 15.6% B, 57.9% C and 18.5% D/7.69min 7.8% A, 70.9% C and 21.3% D/7.99 min 4% A, 36.3% C and 59.7% D/8.68 min 10%A, 90% C/10.2 min 10% A and 90% C.

One sample run took 10.2 min at a flow rate of 0.7 ml per min. During the run the column was heated to 50°C. A fluorescence detector (Acquity UPLC Photodiode Array eλ Detector, Waters, Eschborn, Germany) detected the AQC-derivatized AAs at an excitation wavelength of 266 nm and emission wavelength of 473nm. A mixture of standards with different concentrations were used for the quantification with the Empower Pro Software (Waters, USA).

#### 2.3.3 Carbohydrates

Glucose, fructose and sucrose were analyzed by enzymatic assays, and raw data were processed by the software GEN5 (BioTek, Bad Friedrichshall, Germany) and the Synergy HT Microplate Reader (BioTek, Bad Friedrichshall, Germany). 5 µl of root exudate sample were mixed with 295 µl reaction buffer containing 100 mM imidazol-HCl pH 6.9, 5 mM MgCl_2_, 0.06% (w/v) ATP and 0.075% (w/v) NAD. Then, 0.5 U glucose-6-P dehydrogenase was added to remove internal hexose-P and the baseline was measured at 340 nm (extinction maximum of NADH). In three subsequent reactions, glucose, fructose and sucrose were measured by adding 0.5 U of hexokinase (glucose to glucose-6-P and fructose to fructose-6-P), 0.175 U phosphoglucoisomerase (fructose-6-P to glucose-6-P) and 30 U invertase (sucrose to glucose and fructose) and detecting conversion of NAD^+^ to NADH at 340 nm during the respective enzymatic reactions. Using reaction parameters and the molecular extinction coefficient of NADH_340nm_ = 6.2 mM^-1^ cm^-1^, sugar concentrations were calculated by the software GEN5 (BioTek, Bad Friedrichshall, Germany).

#### 2.3.4 Organic acids

Organic acids were separated and detected using an ion chromatography system with a conductivity detector (Dionex, ThermoFisher Scientific, Germany) connected to a triple quadrupole mass spectrometer QQQ6490 (Agilent Technologies, Waldbronn, Germany). Metabolite separation was done on a high capacity ion exchange column (AS11-HC, 250 x 2 mm), which was connected to a guard column of the same material (AG 11-HC, 10 x 2 mm) and an ATC-1 anion trap column placed between eluents and separation columns to remove the anions in the solution. The Gradient was accomplished as described in Ghaffari et al. (2016). ESI-MS/MS analysis in the negative ionization mode was performed with following parameters: Desolvation at 250°C, N gas flow of 720 l per hour, heater temperature of 250°C, capillary voltage of 3.5 KV and different dwell times between 40 s and 200 s. Collision energy was different among the compounds ranging between 6 and 50 for different masses. Within a span of 1 amu deprotonated ions [M-H]- were monitored. Individual compounds were identified accurately by Multiple Reactions Monitoring allowing to minimize parallel monitoring and to enhance the sensitivity. Authentic standards at different concentrations (Sigma-Aldrich, Darmstadt, Germany) were used for quantification.

#### 2.3.5 Non-targeted metabolite profiling and annotation

Liquid chromatography-electrospray ionization-quadrupole-time of flight-mass spectrometry (LC-ESI-Q-ToF-MS) measurements were based on Böttcher et al. (2009) with some modifications: Chromatographic separations as described using a water/acetonitrile gradient with formic acid and a C18 column. The instrument settings for the Q-ToF-MS contained the following deviations from Böttcher et al. (negative mode and deviating values for positive mode in brackets): mass range: from m/z 90 (50) to 1000; capillary voltage, 4000 V (5000 V); quadrupole ion energy, -5 eV (3 eV); collision energy, -7 eV (3 eV); collision radio frequency, stepping 150/350 Vpp (200/300 Vpp), timing 50/50.

For the acquisition of collision-induced dissociation (CID) mass spectra, the same settings as above were used with additional settings for data dependent acquisition (AutoMSMS): Mode, CID; intensity threshold, 600; number of precursors, 3; precursor background subtraction, on; active exclusion, on after 3 spectra, release after 1 min; smart exclusion, on, 5x; isolation and fragmentation settings, size and charge dependent; width, 3-15 m/z; collision energy, 10-70 eV; charge states included, 1z, 2z, 3z.

The LC-Q-ToF-MS data were processed with Bruker Compass MetaboScape Mass Spectrometry Software, Version 4.0.0 (Bruker Daltonik, Bremen, Germany). Mass recalibration, peak picking, peak alignment, region complete feature extraction, and grouping of isotopes, adduct and charge states was performed with the T- ReX algorithm in Metaboscape. Settings: Peak detection: intensity threshold, 1500 counts; minimum peak length, 7 spectra; feature signal, intensity. Minimum peak length for recursive feature extraction, 3 spectra. Retention time range, 0– 18 min. Mass range, 90 (50) – 1,000 m/z. MSMS import method, average, grouped by collision energy. Ion deconvolution: Extracted-ion chromatogram correlation, 0.8; primary ion, [M+H]+, seed ions, [M+Na]+, [M+K]+, [M+NH4]+; common ions, [M+H- H2O]+ and T- ReX-Recalibration Auto- Detect. Feature filters: Minimum number of samples: present in 3 samples, group filter: present in at least 50% of at least one group.

For annotation, several approaches were combined: First, all fragment spectra were matched in a parallel search against the following databases: NIST17 (The NIST Mass Spectrometry Data Center, U.S. Department of Commerce), MoNa (https://mona.fiehnlab.ucdavis.edu/), ReSpect (Sawada et al., 2012), Riken public databases http://prime.psc.riken.jp/compms/msdial/main.html#MSP), Mass Bank EU (https://massbank.eu/MassBank/Index), GNPS (https://gnps.ucsd.edu/) and an internal database with masses, spectra and retention times of commercial standards (Döll, unpublished), using the MetaboScape “Annotate with Analyte List”- and Spectral Library Search function [Parameters: Filter: exact match of data base entry to precursor mass; tolerances (narrow wide): m/z 10– 30 mDa, mSigma 20– 100, MS/MS score 900– 800]. Afterwards, we performed a manual annotation of compounds already described in literature and in the KNApSAcK database of the four species, with special regard to the top 100 largest peaks in each species and cultivation type. Confidence of annotation: Level 1 = mass and retention time (+CID spectrum, if available) of a commercial or synthesized standard; Level 2 = good evidence from mass spectrum and literature, but no standard, Level 3 = possible structure.

Processing of raw data obtained from non-targeted metabolite profiling: Since non-targeted metabolite profiling of all samples could not be performed consecutively, feature tables of the field and the hydroponic sampling had to be combined in order to allow comparable data evaluation. To do so, features were adopted as identical when in both datasets differences in mass-to-charge (m/z) values and retention time (RT) were< ± 0.01 and< ± 0.3, respectively. Then, features showing a mean height >500 in “water blanks” (containing Micropur^®^ forte in the field root exudate sampling experiment) and in “in-field blanks” were removed from the dataset. Remaining features were regarded as “present” when they were >1000 in height in more than half of a species´ samples collected in the field or >3000 in height in more than half of the hydroponic samples of a species. This threefold difference in thresholds was required, because samples from hydroponic collection were threefold higher concentrated than samples from the field (i.e. field samples were resuspended in 0.75 µl 50% methanol per mg root dry weight while hydroponic samples were resuspended in 0.25 µl 50% methanol per mg root dry weight).

Note: Total C, AAs, carbohydrates and organic acids were quantified in 9-11 individual replicates per field-grown species and in 18-21 replicates per hydroponically cultivated species. In non-targeted metabolite analysis, the same number of field-derived replicates but, due to sample loss, only 3-4 replicates from the hydroponic system were studied.

### 2.4 Statistical analysis

R version 4.1.2 (R Core Team, 2021) was used for statistical analyses. In order to evaluate differences in the recovery of total C and primary metabolites (sugars, AAs, organic acids) in root exudates between the cultivation conditions (“FIELD” vs. “HYDRO”), we fitted the following linear model using the “lm” function:


(model 1) $$Y_{ij}=\mu +\;s_{i}+\;c_{j}+\;{\left(sc\right)}_{ij}+e_{ij}$$

*Y_ij_ …* recovery of total C or a primary metabolite in exudates of the *i* ^th^ species under the *j* ^th^ cultivation condition,

*µ*… intercept term (µ=0),

*s_i_ …* effect of the *i* ^th^ species treated as fixed effect,

*c_j_ …* effect of the *j* ^th^ cultivation condition treated as fixed effect,

*(sc)_ij …_* species-by-cultivation condition interaction treated as fixed effect,

*e_ij_ …* random errors.

In this model, position effects that only occur in the field but not in the hydroponic experiment (due to regular randomization of the pots), are included in *c*. Normality was evaluated based on the residuals (extracted by “resid” function) with diagnostic plots as described by Kozak and Piepho (2018) and data were log-transformed when necessary. With the emmeans package version 1.7.0 (Lenth, 2021) significant differences were computed based on the estimated means (calculated by “emmeans” function) using the “contrasts” function (method=“pairwise”, adjust=“fdr”, alpha=0.05).

In order to calculate how much of the variance in the datasets for secondary and primary metabolites was explained by the cultivation condition (*Var[C]*), we first created a binary presence/absence dataset for features detected by non-targeted LC-MS/MS (see 2.3), while data for primary metabolites were imputed as quantitatively measured values. Then, the data were subset for species. With the “cca” function of the vegan package version 2.5-7 (Oksanen et al., 2020), we computed the variance explained by the cultivation condition (“FIELD” vs. “HYDRO”) in the individual datasets for species by constrained correspondence analysis on a *χ^2^*-transformed data matrix (Legendre and Legendre, 2012).

Principal component analyses (PCA) were performed to visualize global differences between the two cultivation conditions and the four species for primary and secondary metabolites with the stats package version 4.1.2, “prcomp” function (R Core Team, 2021). Features detected by non-targeted LC-MS/MS were in a binary presence/absence dataset, while for primary metabolites we imputed the total recovery in the sampling solution (2.2.) and scaled data prior to PCA by building z-scores *via*


(equation 1) $$z_{i}=\frac{x_{i}-\;m\;}{sd}$$

*z_i_ …* z-score of the *i* ^th^ recovery of a metabolite,

*x_i_ … i* ^th^ recovery of a metabolite,

*m …* mean recovery of a metabolite,

*sd …* standard deviation of the recovery of a metabolite.

## 3 Results

### 3.1 Total carbon and chemical richness of primary and secondary metabolites in root exudates of cover crops under different cultivation systems

**Figure 1:**
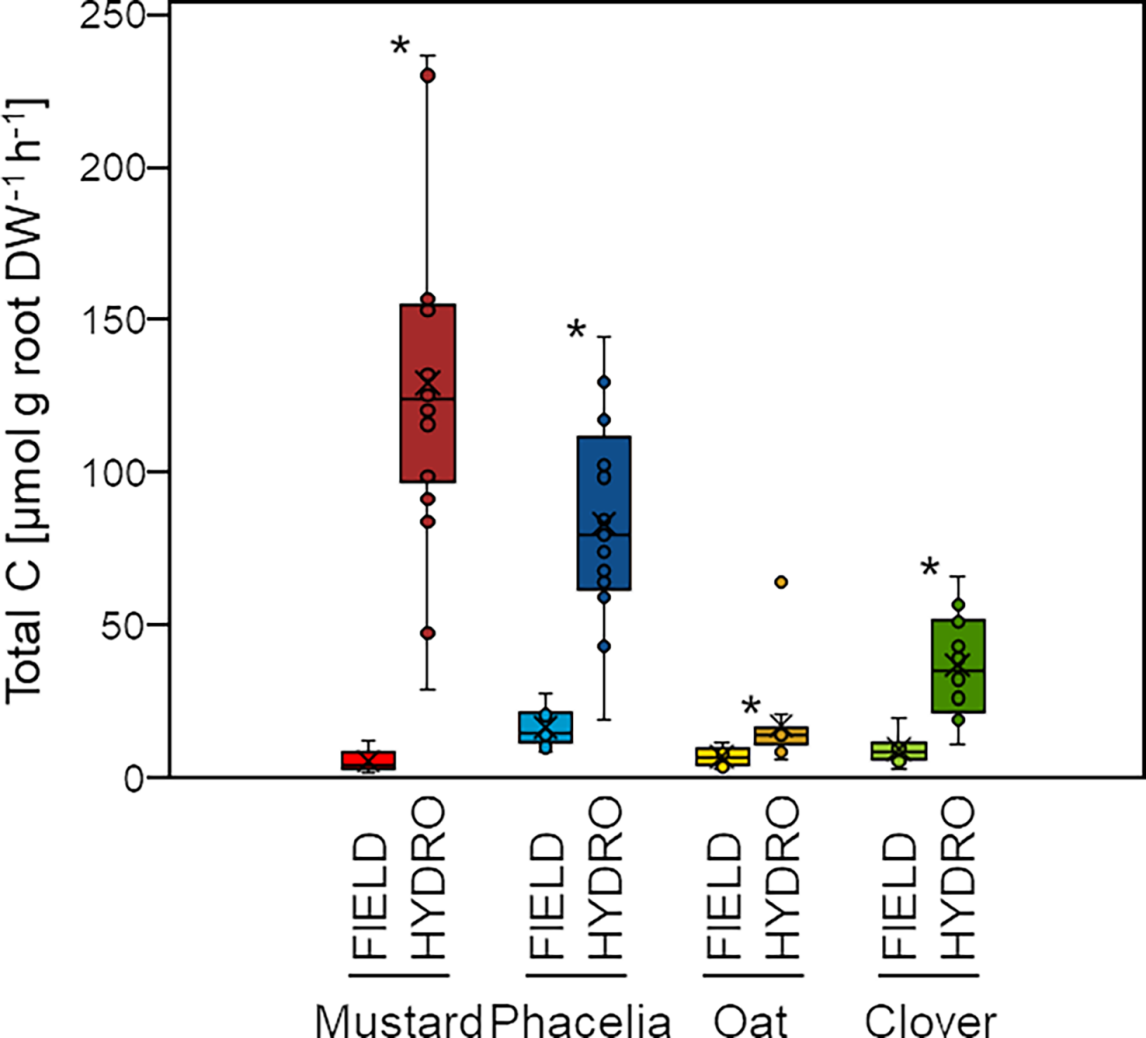
Recovery of total carbon in root exudates of cover crop roots in the field (FIELD) and in hydroponic culture (HYDRO). Boxes show median, first and third quartile, whiskers show minimum and maximum; a value is shown as outlier when it exceeds/undercuts the third/first quartile by more than 1.5x the interquartile range (n=9-11 [FIELD], n=18-21 [HYDRO]). Asterisks mark significant differences between FIELD and HYDRO within one species according to differences in estimated means extracted from model 1 at the 5% level of significance.

We first investigated whether root release of total C, the number of semi-polar secondary metabolites, and the number and quantity of a set of primary metabolites differed when cover crops were cultivated in the field or in hydroponics. In hydroponics, we recovered the largest quantity of total C on a root biomass basis in mustard, followed by phacelia, clover and oat. The same ranking was found in field-collected exudates for phacelia, clover and oat, whereas mustard showed the lowest quantity of C in the field (**Figure 1**). However, in all species we collected larger quantities of total C when plants were grown in hydroponics. In mustard ~27x more C was recovered compared to the field; in phacelia, oat and clover we found at least ~5x, ~2.5x or ~4x more C, respectively.

**Figure 2:**
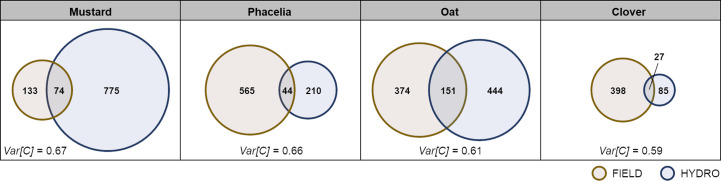
Feature presence of secondary metabolites in root exudates of cover crops grown in the field or hydroponically. Venn diagrams show numbers of unique semi-polar features detected by an untargeted LC-MS/MS approach in root exudates of mustard, phacelia, oat and clover sampled in the field (FIELD) or hydroponically (HYDRO) and features being present in both cultivation systems within the overlapping part; n=9-11 [FIELD], n=3-4 [HYDRO]. “*Var[C]*” indicates the variation explained by the cultivation conditions (FIELD vs. HYDRO) calculated on a presence/absence matrix for secondary metabolites in individual samples by constrained correspondence analysis.

Non-targeted LC-MS/MS-based metabolite profiling was used to analyse profiles of semi-polar secondary metabolites. In hydroponics, mustard exudates contained with 849 the largest number of features, being 1.4, 3.3 and 7.6x higher than those in oat, phacelia and clover, respectively, while under field conditions, mustard exudates had with 207 the lowest number of features, being only 34% of those found in phacelia - the species with the highest feature count (**Figure 2**). With exception of mustard, all species allowed recovery of similar or a severalfold larger number of features when exudates were collected in the field: Chemical richness in oat was comparable between field and hydroponics, while in phacelia and clover it was 2.4-fold or 3.8-fold higher, respectively, when cultivated in the field (**Figure 2**).

For primary metabolites, we performed a targeted screen of a set of 28 primary metabolites, comprising 20 amino acids, 5 organic acids and 3 sugars. Although this number does not represent the diversity of primary metabolites, this approach came with the advantage of quantitative assessment. From the 28 primary metabolites, we found 18 in mustard and phacelia, 21 in oat and 13 in clover (**Table 1**). Detected primary metabolites were - in number - most abundant in exudates of mustard collected hydroponically, followed by oat, clover and phacelia. In contrast, under field conditions the largest number of primary metabolites was recovered from phacelia, being >5x more than in mustard exudates, which showed the lowest primary metabolite number in the field (**Table 1**). Comparing both cultivation systems, the number of primary metabolites in mustard and oat was 5.6x or 1.7x higher in hydroponics, while in clover there was no difference among cultivation systems and in phacelia 4x more primary metabolites were recovered in the field (**Table 1**). On a quantitative basis, sugars were the dominant group of considered primary metabolites in hydroponically-grown mustard and accounted in total for >200 nmol C root DW^-1^ h^-1^. This equates to >50 µg C and represents a share of >3% in the total C mass of 1.5 mg in mustard exudates (**Figure 1**; **Table 1**). Whereas AAs contributed only by 1% to total C in mustard, they made the majority of primary metabolites in oat exudates with >12% of the total C mass of 0.2 mg. In clover exudates, sucrose was very prominent and contributed by ~6% to total C (0.4 mg), while other primary metabolites shared in sum ~1% of total C. In phacelia, all investigated primary metabolites made in total less than 1.5% of C, which was 1 mg, in root exudates. This comparison suggests that especially in mustard and phacelia large amounts of other C-containing compounds were released, such as sugar alcohols as well as secondary metabolites that could not be assessed quantitatively. In field-collected exudates from phacelia, organic acids, sugars or AAs shared 6, 9.2 or 15.8%, respectively, of the total C mass of 0.2 mg (**Figure 1**; **Table 1**). Also in clover, 8% of total C mass of 0.1 mg was contributed by organic acids and >10% by sugars as well as AAs. While in oat organic acids and sugars were recovered to some extent, mustard exudates contained only five of the quantified primary metabolites at all.

**Table 1:** Exudation rates of primary metabolites by cover crop roots in the field (FIELD) and in hydroponic culture (HYDRO).

	Mustard		Phacelia		Oat		Clover	
	FIELD	HYDRO	FIELD	HYDRO	FIELD	HYDRO	FIELD	HYDRO
Metabolites [nmol g^-1^ root DW h^-1^]								
Organic acids								
Citrate	n.d.	8.1 ± 5.7	17.5 ± 15.2	1.1 ± 1.0 *	2.4 ± 3.5	n.d.	73.9 ± 71.5	0.7 ± 0.8 *
Fumarate	n.d.	n.d.	137.0 ± 98.3	n.d.	n.d.	n.d.	9.2 ± 2.1	n.d.
Malate	n.d.	16.3 ± 13.1	67.0 ± 38.3	n.d.	8.6 ± 8.6	19.3 ± 6.8 *	49.3 ± 47.3	n.d.
Succinate	n.d.	n.d.	6.1 ± 3.5	n.d.	1.6 ± 1.0	n.d.	5.6 ± 4.1	n.d.
Carbohydrates								
Glucose	n.d.	222.8 ± 143.7	50.1 ± 36.2	n.d.	n.d.	n.d.	52.2 ± 31.7	52.4 ± 32.8
Fructose	22.9 ± 14.2	153.6 ± 113.3 *	43.6 ± 29.6	68.5 ± 59.3	24.1 ± 21.7	n.d.	50.0 ± 32.9	n.d.
Sucrose	n.d.	161.0 ± 114.9	73.7 ± 41.0	56.6 ± 43.3	10.6 ± 5.9	17.8 ± 12.5	24.3 ± 18.1	170.4 ± 134.9 *
Amino acids								
Ala	23.7 ± 8.8	52.0 ± 35.3	46.1 ± 25.7	n.d.	30.9 ± 13.2	54.7 ± 15.4	29.9 ± 21.0	17.7 ± 9.3
Arg	n.d.	n.d.	n.d.	n.d.	n.d.	23.4 ± 9.0	n.d.	n.d.
Asn	n.d.	n.d.	n.d.	n.d.	n.d.	n.d.	56.7 ± 45.7	19.1 ± 6.4
Asp	n.d.	n.d.	27.1 ± 9.9	n.d.	22.1 ± 4.8	21.4 ± 8.3	n.d.	n.d.
Gaba	n.d.	62.3 ± 25.0	n.d.	25.8 ± 23.6	n.d.	n.d.	n.d.	n.d.
Glu	n.d.	20.3 ± 12.4	27.3 ± 8.4	n.d.	n.d.	37.1 ± 9.7	n.d.	20.4 ± 23.5
Gln	31.8 ± 19.5	n.d.	234.4 ± 187.5	n.d.	76.4 ± 48.3	n.d.	117.5 ± 113.5	n.d.
Gly	n.d.	15.2 ± 7.1	34.1 ± 17.5	n.d.	n.d.	51.0 ± 15.1	n.d.	n.d.
Ile	n.d.	n.d.	n.d.	n.d.	n.d.	15.6 ± 5.5	n.d.	n.d.
Leu	n.d.	20.0 ± 11.4	32.5 ± 24.6	n.d.	19.1 ± 4.5	40.1 ± 12.2 *	n.d.	n.d.
Lys	n.d.	14.7 ± 8.6	n.d.	n.d.	n.d.	23.7 ± 9.9	n.d.	n.d.
Met	25.2 ± 10.0	18.7 ± 12.8	n.d.	n.d.	n.d.	26.4 ± 10.1	n.d.	18.8 ± 6.6
Phe	n.d.	n.d.	n.d.	n.d.	n.d.	13.7 ± 4.0	n.d.	n.d.
Pro	n.d.	12.9 ± 4.9	n.d.	n.d.	n.d.	13.6 ± 4.0	n.d.	n.d.
Ser	n.d.	13.2 ± 6.4	45.6 ± 25.4	n.d.	n.d.	20.4 ± 6.4	n.d.	n.d.
Thr	n.d.	16.7 ± 6.5	26.7 ± 9.4	n.d.	n.d.	19.3 ± 5.4	n.d.	n.d.
Tyr	45.9 ± 39.8	23.9 ± 19.9	32.8 ± 34.9	n.d.	33.8 ± 15.9	29.7 ± 9.9	n.d.	15.0 ± 11.7
Val	n.d.	17.1 ± 10.0	28.4 ± 20.0	n.d.	19.7 ± 5.2	32.4 ± 10.0 *	n.d.	n.d.
*Var[C]*	0.40		0.45		0.57		0.36	

Values show means ± SD (n=9-11 [FIELD], n=18-21 [HYDRO]). Asterisks mark significant differences between FIELD and HYDRO within one species according to differences in estimated means extracted from model 1 at the 5% level of significance. n.d.=not detected in more than 50% of the samples. Note: Isocitrate and the AAs His and Orn could not be detected in root exudates of any species. *“Var[C]”* indicates the variation explained by the cultivation conditions (FIELD vs. HYDRO) calculated on quantitative data for primary metabolites in individual samples by constrained correspondence analysis.

In order to determine if the cultivation condition has a larger impact on the recovery of secondary metabolites or of quantitatively assessed primary metabolites, we calculated the variance explained by the cultivation condition in the respective datasets, separately for every cover crop. For secondary metabolites, *Var[C]* of mustard was highest among all species with 67% and lowest in oat and clover with 61% and 59%, respectively (**Figure 2**). There were stronger differences among the species in the datasets for primary metabolites. While in clover only 36% of the variance in primary metabolites was explained by the cultivation condition, *Var[C]* in oat was 57% (**Table 1**). Overall, the *Var[C]* was higher for secondary than for primary metabolites, and differences in *Var[C]* among species were lower for secondary metabolites.

Taken together, we found that in mustard a larger quantity of root exudates recovered in hydroponics was associated with a higher chemical richness of both, determined primary and secondary metabolites. In oat, C recovery and the number of metabolites were comparatively less affected by the cultivation condition, while phacelia and clover showed a larger quantity of total C in hydroponics. Nonetheless, those latter two species showed higher chemical richness in field-collected exudates.

### 3.2 Diversity of secondary and primary metabolites in different cultivation conditions

**Figure 3:**
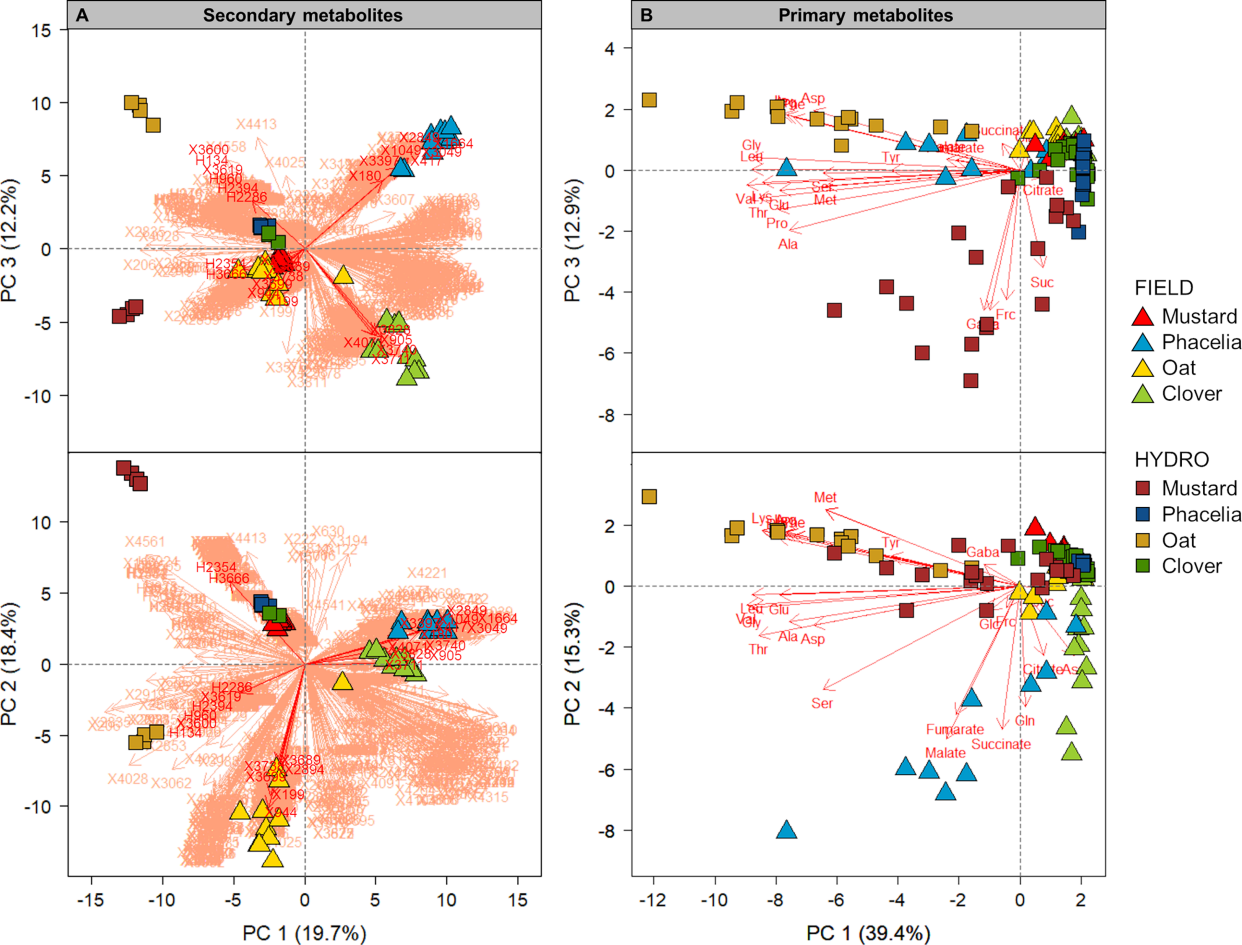
Diversity of primary and secondary metabolites in root exudates of mustard, phacelia, oat and clover grown in the field (FIELD) or hydroponically (HYDRO). Principal component analysis of secondary **(A)** and primary **(B)** metabolites. For primary metabolites, recovery as given in **Table 1** was used as input data and scaled prior to PCA analysis; for secondary metabolites a binary presence/absence matrix of semi-polar features detected by an untargeted approach was imputed without scaling. Plots show the principal components (PC) 1, 2 and 3 and the variance explained by them in brackets. Red arrows represent eigenvectors of the covariance matrices of primary or secondary metabolite data. In **Figure 3A** the eigenvectors of level 1-3-annotated compounds mentioned in section 3.2 are highlighted.

In order to address the question which metabolites are most discriminative for species and growth conditions, we used PCA on secondary and primary metabolite data. Although secondary metabolites were not determined in quantity, it appeared that their sheer number led to a largely distinct separation of species and cultivation conditions in a PCA when using a simple binary presence/absence dataset (**Figure 3A**). Hydroponic samples of mustard and oat could be clearly separated along PC2 and PC3, which explained 18.4% and 12.2%, respectively, of the variance in the dataset, while phacelia and clover samples could not be separated along the first three PCs as ~1/4 of the features detected in clover were identical to those of phacelia. According to eigenvectors, the separation of Oat_HYDRO was driven by ~300 metabolites, among them were the most detailed annotated compounds: S(8-O-4)esculetin (X3619), which could be annotated at level 2, several level 3-annotated triterpenoid saponins (H2286, H2394) and methylanthranilic acid-related compounds (H960, X3600) and level 2-annotated avenic acid (H134; **Figure 3A**). In mustard, directions of eigenvectors of only two metabolites fitted the position of the Mustard_HYDRO group in the PC1 vs. PC3 plot, namely H3666 and H2354, where the latter had the 11^th^ highest peak height among all hydro features in mustard (**Table S1**). However, both of them could not be annotated. Field samples grouped by species for mustard and oat along PC2, while PC3 was necessary to separate phacelia and clover (**Figure 3A**). Oat exudates were mainly separated by ~145 compounds, among them level 1-annotated 2’-deoxyadenosine (X199), tyrosyl-isoleucine (X2894) and jasmonic acid (X3699) and different putative saponins (X3738, X944, X3689). Phacelia_FIELD grouped based on ~100 compounds, e.g. the level 1 annotated quinoline (X417), a glycyrrhizin-like triterpenoid saponin (X1049), coumarin (X3397), niacinamide (X180), level 2 determined dopamine glucuronide (X2849), an unknown steroid (X1664) and a triterpenoid (X3049). Clover_FIELD separated mainly based on ~200 compounds, where especially phenylpropanoids like biochanin A (X4071), formononetin (X3828), putative trifolirhizin6’-O-malonate (X3740), genistein (X3711), kaempferol (X905) drove the separation. We observed a separation of hydroponically and field-sampled exudates along PC1 and PC2. There, eigenvectors pointing into the fourth quadrant of the PC1 vs. PC2 coordinate system belong, as far as annotation was possible, to putative “lipids and lipid-like molecules” (according to the classification system described in 3.3.).

Similar to our observations for secondary metabolites, we found that primary metabolite patterns in hydroponically sampled root exudates of mustard separated from those of oat along PC3, which explained 12.9% of the variance in the dataset (**Figure 3B**). As indicated by eigenvectors, the sugars glucose (Glc), fructose (Frc) and sucrose (Suc) together with the AA Gaba were mainly contributing to the separation of mustard, while Tyr, Lys, Arg, Phe and Ile drove the separation of oat. Hydroponic phacelia and clover exudates could not be separated along the first three PCs and grouped closely together. Other than in the PCA of secondary metabolites, field-collected exudates of the different species separated in a less pronounced manner than hydroponic exudates along PC1 and PC2, which explained 39.4% and 15.3%, respectively, of the variance in the primary metabolite data. The organic acids fumarate, malate and citrate were discriminative for phacelia samples, while Asn made a strong contribution in discriminating clover exudates. Altogether, secondary metabolites allowed a more distinct separation by species and cultivation condition than quantitative data on primary metabolites.

### 3.3 Chemical classification of secondary metabolites in root exudates from different growth conditions

**Figure 4:**
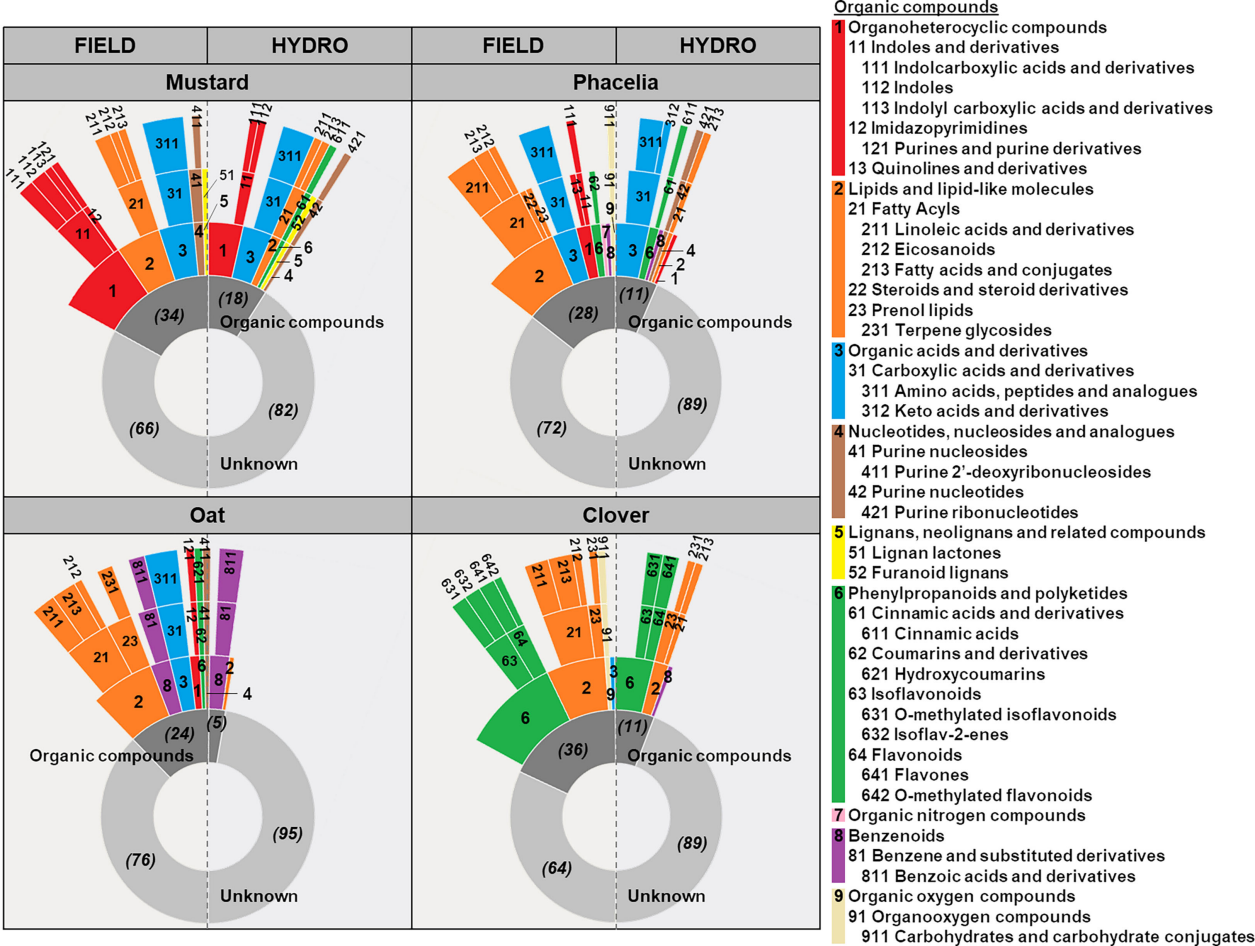
Putative chemical classification of the 100 most abundant features in root exudates of mustard, phacelia, oat and clover grown in the field (FIELD) or hydroponically (HYDRO). Features were sorted by peak height and the top 100 in either growth condition are shown in **Table S1**. Those were classified using the ClassyFire tool (http://classyfire.wishartlab.com; Djoumbou Feunang et al. (2016)) by their *kingdom* and *superclass*, when compounds could be putatively annotated by level 3, and by *kingdom*, *superclass*, *class* and *subclass*, when putative annotation could be made on level 1 and 2 (see **Table S1**). Figure legend shows *superclass* on rank 1 (e.g. 1 Organoheterocyclic compounds), *class* on rank 2 (e.g. 11 Indoles and derivatives) and *subclass* on rank 3 (e.g. 111 Indolcarboxylic acids and derivatives). Features without annotation were classified as “Unknown”. The actual number of features classified as “Organic compounds” or “Unknowns” in every species and cultivation condition is given in brackets.

We identified a number of secondary metabolites as discriminative for most species x cultivation condition groups, ranging from ~100 (Phacelia_FIELD) to ~300 (Oat_HYDRO) as shown in section 3.2. In the following we assigned secondary metabolites in root exudates of each species under either cultivation condition to dominant chemical classes considering the 100 compounds with highest peaks per species and cultivation condition (**Table S1**). Putatively identified compounds were sorted into their chemical classes using the ClassyFire tool (http://classyfire.wishartlab.com; Djoumbou Feunang et al. (2016)). However, annotated compounds represented at maximum about one third of all features (clover and mustard, FIELD), while the rest of the features had to be classified as “unknown” (**Figure 4**).

In mustard and clover, we found an overall similar pattern of the main chemical *superclasses* in field and hydroponic cultivation (**Figure 4**). Although in general less compounds could be annotated in hydroponics, organic compounds assigned to the dominant *superclass* held a similar share in hydroponics and under field conditions. In clover exudates, about half of the compounds were tentatively classified as “phenylpropanoids and polyketides” in both cultivation systems and in mustard “organoheterocyclic compounds” had a similar portion. The contributions of metabolites in the further *superclasses* “lipids and lipid-like molecules” in both species or “organic acids and derivatives” in mustard were more variable among growth conditions and metabolites in less-prominent *superclasses* were often only present in one cultivation system. The number of compounds assigned to individual chemical *superclasses* in phacelia was more strongly affected by the cultivation conditions. Here, metabolites classified as “lipids and lipid-like molecules” held a share of about 50% of annotated compounds in the field, while in hydroponics most compounds were “organic acids and derivatives”. In oat, we found about 50% of putative “lipids and lipid-like molecules” in the field exudates as well, while in hydroponics metabolites of the “benzenoids” were most abundant. However, as we could only annotate five of the features in hydroponically sampled exudates, comparison of both cultivation conditions in oat was difficult, and at *class* and *subclass* levels chemical identity of the spectrum of annotated compounds became much more distinct in all species.

Comparing the tentative chemical classification of metabolites among species, we found highly characteristic patterns in all cover crops. Mustard exudates contained especially compounds assigned to “organoheterocyclic compounds” out of which primarily indole-compounds were annotated at level 1 (**Figure 4**; **Table S1**). Additionally, level 1- and level 2-annotated di- and tripeptides contributed by 15-22% to putatively classified compounds in mustard. In clover, strong emphasis was on “phenylpropanoids and polyketides” of the *classes* “isoflavonoids” and “flavonoids”. Irrespective of the cultivation system, biochanin A had by far the highest peaks in root exudates of clover, but also formononetin and kaempferol were among the top ten compounds in either growth condition. In exudates of both, mustard and clover, we found also a considerable number of “lipid and lipid-like molecules” from the “fatty acyls” *class*, while further chemical classes made only at most 20% of classified organic compounds. In phacelia and oat “lipid and lipid-like molecules” were most prominent under field conditions, but additionally there were compounds of six and five, respectively, further putative chemical *superclasses* contributing in total to about one half to the classified compounds in those species. “Organic acids and derivatives” were prominent in both species and, as observed in mustard, di- and tripeptides made the largest share in that superclass. While in phacelia further metabolites like cinnamic acid, coumarin, quinoline, pantothenic acid and adenosine monophosphate were identified, a smaller number of compounds could be annotated in oat. Here, at least level 2 annotation suggested especially methylanthranilic acid and several related “benzenoids” to be constitutively released under both cultivation conditions. In general, we observed that each species showed characteristic metabolites (e.g. indole-compounds in mustard or isoflavonoids and flavonoids in clover).

## 4 Discussion

Sampling of root exudates under field- or field-like conditions is laborious but expected to yield more realistic patterns of root-released metabolites than the often-used hydroponic sampling method, since in soils exudation is subject to the influence of physical, chemical and biological factors, such as mechanical stimulation of roots by soil particles or their interaction with soil organisms. On the other hand, root-washings from field-grown plants come with the drawback of larger variability, as imposed by stress responses and root lesions (Oburger and Jones, 2018; Williams et al., 2021). Nonetheless, under defined environmental conditions or stimuli, like nutrient deficiencies or heavy metal stress, at least some of the same key metabolites can be recovered irrespective of whether plants were cultivated in soil or hydroponic conditions (Kawasaki et al., 2018; Rosenkranz et al., 2021; Sarashgi et al., 2021). It is thus important to understand how the cultivation and collection conditions affect C release by roots globally, as well as which classes of root-exuded metabolites are subject to variation through growth conditions and if there are commonalities across species. Here, we place emphasis on identifying compounds and compound classes, which are discriminative for the cultivation in the field vs. hydroponics for four different cover crop species and test whether the pattern of a large set of non-quantified secondary metabolites is more characteristic for plant species and cultivation conditions than that of 28 quantitatively determined primary metabolites.

### 4.1 The recovery of total carbon is not indicative for the diversity of metabolites in root exudates

When we compared root exudates sampled in the field and in hydroponic growth conditions, the recovery rate of total C in HYDRO was much higher in each of the cover crops (**Figure 1**). However, due to plant cultivation conditions and methodological constraints a direct comparison of absolute values of C recovery rates between HYDRO and FIELD is biased by a number of factors: i) the microbial density and thus the potential for microbial degradation might still be higher for exudates from field-grown plants, (ii) root damages caused during the washing procedure may cause leakage of intracellular metabolites into the sampling solution in the field (Oburger and Jones, 2018), (iii) suboptimal nutritional conditions and higher fluctuations in temperature and light intensity in the field (Langstroff et al., 2021) may cause lower photosynthesis rates than in hydroponics (Vialet-Chabrand et al., 2017), and (iv) the longer sampling time in hydroponics may increase the total C recovery. We aimed to reduce microbial growth by treating field-sampled exudates with Micropur^®^. However, this agent may not only suppress microbial degradation but also reduce metabolite secretion by roots. During a short sampling period of two hours Micropur^®^ can decrease metabolite recovery in exudates by up to 70% for organic acids and up to 50% for phenylpropanoids (Valentinuzzi et al., 2015). Furthermore, root injuries during washing could not be prevented in field-grown plants (**Figure S1**), so that we had to assume a certain contribution of intracellular metabolites to total exudate-C in field samples. However, at least among the annotated compounds within the top 100 we could not detect any metabolite being exclusively intracellular (**Table S1**). In addition, among all field-grown cover crops, C recovery in mustard - the species with most root lesions (**Figure S1**) - was lowest (**Figure 1**). This suggests that internal stores of metabolites may have been leaking out when roots were rinsed before the sampling begun or that the fine roots have had a reduced exudation capacity due to wounding, resulting in lower C recovery in field-grown plants in our sampling approach. A recent study proposes several days of recovery treatment after root washing to decrease leakage of intracellular metabolites (Williams et al., 2021). Such a lengthy recovery period raises the question to what extent the exudate pattern still reflects that of soil-grown roots. In our study, we desisted from a recovery period in favor of obtaining metabolite profiles mirroring the growth situation in the soil (Oburger and Jones, 2018). Taking all these factors together, we envisage an underestimation of total C recovery in root exudates from field- relative to hydroponically-grown plants.

Interestingly, the chemical richness appeared not to be compromised from saving on C but was even higher in exudates from field-grown phacelia or clover despite the weaker total C recovery (**Figure 2**). The presence of a soil environment may have augmented the number and type of recovered metabolites. This will affect exudate secretion processes and even upstream biosynthesis pathways, as shown for example by the soil pH-sensitive release of iron-mobilizing coumarin species in *Arabidopsis thaliana* (Rajniak et al., 2018). In fact, such influential factors may also apply to those species whose metabolite variety increased with C exudation rate. Oat exhibited twofold more C in hydroponics but showed under both cultivation conditions a similar chemical richness albeit of largely different qualitative composition (**Figures 1**, **2**). Together with the inverse relation between metabolite number and exudate-C as observed in phacelia and clover, this observation clearly rejects the hypothesis that the recovery of total C is indicative for the number of metabolic compounds in root exudates.

### 4.2 Specific patterns of secondary metabolites are discriminative for species and growth conditions

A main goal of this study was to identify if non-targeted secondary metabolites or a smaller set of quantitatively assessed primary metabolites are more affected by species and cultivation conditions. We addressed this question in two ways, namely by calculating the variances in datasets for secondary and primary metabolites and by evaluating groupings for secondary or primary metabolites in PCAs in dependence of species or cultivation conditions. We found that *Var[C]* was higher in datasets of secondary than of primary metabolites (**Table 1**; **Figure 2**) and the PCA discriminated more sharply between species and cultivation method for secondary than for the limited number of quantified primary metabolites (**Figure 3**). Thus, we conclude that the secondary metabolite composition is more characteristic for species and growth conditions than that of primary metabolites, even though the latter builds on quantitative values. This is partly in accordance with a study of Dietz et al. (2020), who analyzed 285 polar features detected by gas chromatography-MS in ten grassland species at three sites in Germany and showed that the chemical composition of semi-polar metabolites was more subject to the plant species, while the exudation profile of polar metabolites was more affected by the growth environment. Nevertheless, employing PCA we observed that several primary metabolites were explanatory for the separation of Phacelia_FIELD, Mustard_HYDRO or Oat_HYDRO from the other combinations of species and growth conditions (**Figure 3B**). For example, the organic acids fumarate, malate and citrate were indicative for Phacelia_FIELD, as that species showed the largest amount of organic acids in the field. However, also in clover the abundance of organic acids in field-sampled exudates was higher than in hydroponics, and in oat at least the diversity of organic acids increased in the field (**Table 1**). Thus, field conditions appeared to stimulate the release of a larger number and quantity of organic acids. Organic acid exudation can play a role in aluminum detoxification at low pH or improve the acquisition of P and Fe (Sharma et al., 2016; Upadhyay et al., 2019). The soil at the Asendorf field site had a pH of ~6.25 in the uppermost 30 cm, i.e. a soil pH that excludes aluminum toxicity (Sullivan and Gadd, 2019). We thus anticipate organic acids in root exudates as a strategy to improve nutrient acquisition. Despite an elevated root:shoot biomass ratio (**Table S2**), which is indicative for N or P deficiency (Marschner, 2012), we did not observe visual symptoms of nutrient disorders. Hydroponically-grown mustard and oat were separated from the other species/cultivation conditions by a large number of AAs along PC1 (**Figure 3B**) which was likely driven by better N nutrition in hydroponics as has been also observed in maize (Carvalhais et al., 2011; Zhu et al., 2016). Also, clover exhibited a larger number and quantity of AAs in hydroponics compared to the field (**Table 1**), so that a high recovery of AAs in root exudates appears to be typical for hydroponic growth conditions. Apart from mustard, all other cover crops showed more sugars when grown in the field (**Table 1**). Plants can deliver sugars to plant growth-promoting rhizobacteria, a process that is most likely mediated by transporters of the SWEET family (Hennion et al., 2018). We anticipate that the higher sugar recovery from soil-grown plants (**Table 1**) resulted from an interaction with a more complex microbiome in the field than in hydroponics. Altogether, we conclude that primary metabolite patterns in root exudates reflect general responses to the cultivation conditions and are not discriminative for individual plant species.

### 4.3 Biological functions of species-specific root exudates

The chemical classification of recovered secondary metabolites revealed that every species showed a characteristic pattern of chemical *superclasses* (**Figure 4**). Within the frame of those *superclasses*, metabolite profiles became even more distinct for the cultivation condition in every species at *class* and *subclass* level (**Figures 3A**, **4**). For example, the presence of phenylpropanoids was largely discriminative for clover, even though the number and identity of specific phenylpropanoid compounds recovered under different cultivation conditions varied (**Table S1**). As a legume, clover communicates with rhizobia and mycorrhizal fungi in the rhizosphere (Hassan and Mathesius, 2012). In particular the release of exudates with signaling functions in microbial associations depends on the presence and interaction with the respective microbial community (Olanrewaju et al., 2019), likely explaining the strong increase in metabolite recovery in the field (**Figure 2**). Phenylpropanoids commonly regulate the specific interaction of legumes with their symbionts, but inhibit also root pathogens or the growth of other plants in the surrounding (Hassan and Mathesius, 2012). In our study, we found 20 putative metabolites of the “phenylpropanoid and polyketide” *superclass* among the top 100 compounds in the field, but only eight of the same *superclass* in hydroponics (**Table S1**). This is in accordance with the distinct grouping of Clover_FIELD exudates, while Clover_HYDRO could not be separated from Phacelia_HYDRO and Mustard_FIELD (**Figure 3A**). The flavonoids genistein and daidzein, which are level 1-annotated only in field exudates (**Table S1**), have been shown to induce *nod* genes in *Rhizobium* strains as prerequisite for infection and nodulation of clover plants (Kosslak et al., 1987). In contrast, biochanin A and formononetin appeared to be constitutively present in root exudates regardless of the cultivation conditions (**Table S1**). These latter metabolites are known to stimulate the growth of mycorrhizal hyphae and root colonization of *Trifolium repens* (Nair et al., 1991). However, in another study with *Trifolium pratense* biochanin A, formonentin and genistein were suggested to play a role in biotic stress responses (Dinkins et al., 2021). This emphasizes the high species- and condition-specific action of phenylpropanoids in biotic interactions (Hassan and Mathesius, 2012). In addition, the level 1-annotated phenylpropanoids genistein and kaempferol (**Table S1**) can reduce Fe(III) improving Fe availability (Cesco et al., 2010), as clover relies on elevated Fe supply in order to maintain leghemoglobin formation for biological N_2_ fixation by symbiotic bacteria (Brear et al., 2013). Despite the absence of visual symptoms of nutrient disorders or pathogen infection, clover showed reduced shoot growth in the field compared to hydroponics (**Table S2**). This may be due to an altered physiological status of the plants in response to different temperature, light, nutrient and water supplies, or pathogen pressure.

Compared to clover, whose tentatively annotated root exudates consisted mainly of two chemical *superclasses*, field-sampled exudates of oat and phacelia were much more diverse in their chemical composition (**Figure 4**). We found in exudates of oat and phacelia compounds assigned to six and seven *superclasses*, respectively. Both species had in common a considerable amount of di- and tripeptides, which could be annotated at level 1 only in oat. A similar observation was made in mustard which is in accordance with a study of Strehmel et al. (2014), who recovered large amounts of di- and tripeptides from root exudates of *Arabidopsis thaliana*. These exudates may function as chemotactic agents for microbes (Sourjik, 2004), as signals in plant-plant communication or as N sources for neighbors (Komarova et al., 2008).

Along PC1 there was a clear separation of field- and hydroponically collected root exudates of oat, which was driven by ~145 or ~300 features, respectively (**Figure 3A**). The constitutive recovery of putative “benzenoids” under both cultivation conditions was very characteristic for oat (**Figure 4**). In fact, Poaceae species can synthesize and release benzoxazinones which are precursors of potent allelochemicals (Fomsgaard et al., 2004). The “benzenoids” compounds in our study were level 2-annotated as methylanthranilic acid or related compounds (**Table S1**), but a role of those metabolites in root exudates has, to our knowledge, not been described so far. Phacelia_FIELD was determined by ~100 metabolites, among them the “organoheterocyclic compound” quinoline and the phenylpropanoid coumarin, which were present among the top 100 compounds (**Figure 3A**; **Table S1**). Those compounds have potentially phytotoxic action (Weir et al., 2010; Flamini, 2012). In hydroponics we found cinnamic acid as the top 4 compound (**Table S1**), which is as well an allelopathic substance (Flamini, 2012), but was also described as a highly potent autotoxic compound in cucumber (Ding et al., 2007). Also mustard showed cinnamic acid in larger quantities only in hydroponics (**Table S1**). In these two species one may speculate about an autotoxic function of cinnamic acid in growth regulation of neighbors, but this aspect remains to be further investigated.

Mustard showed the lowest feature count among all species in the field (**Figure 2**). This was most likely due to injured roots (**Figure S1**). Nevertheless, a closer inspection of the recovered compounds reveals that mustard exudates are dominated by metabolites that serve for biotic defense. In this context, particularly striking is the high recovery of compounds of the “indole and derivatives” *class* (**Figure 4**; **Table S1**). We found indole-3-carboxaldehyde and methyl indole-3-carboxylate in either growth condition and detected indole-3-carboxylic acid and 5-methoxyindole specifically in the field. The former three metabolites might be breakdown products of the phytoalexin brassinin (Pedras et al., 2002). Likewise, tryptophan-derived metabolites have been detected also in root exudates of *Arabidopsis thaliana* (Strehmel et al., 2014) and proposed to act in pathogen defense (Bednarek et al., 2005). Also, level 3 annotation tentatively suspects the release of phytoalexins (brassilexin, Pedras and Minic, 2014; sinalbin A, Pedras and Zaharia, 2000) and an indole glucosinolate (**Table S1**). The latter represent highly potent allelopathic substances common to members of the Brassicaceae (Flamini, 2012).

Among the top 100 compounds, “lipids and lipid-like molecules” were very prominent in root exudates of all field-grown plants and were highly abundant among the compounds driving the separation from hydroponically sampled exudates in all species (**Figures 3A**, **4**). In general, they act in signaling and defense responses (Lim et al., 2017; Wen et al., 2021), as indicated for the potent antifungal triterpenoid saponins (Osbourn, 2003), which were level 2-annotated in oat exudates here (**Table S1**), and are part of mucilage improving root penetration through the soil (Holz et al., 2018). In addition, they have been shown to be released in larger quantities under water deficit in oilseed rape (Svenningsson et al., 1990). The dry conditions during our field experiment may have favored the recovery of “lipids and lipid-like molecules” in the present study (**Figures 3A**, **4**). The reduced growth of all species in the field at a similar developmental stage as in hydroponics (**Table S2**) may indicate an altered physiological status in response to different environmental conditions like temperature, light, nutrient and water supplies, or pest and pathogen pressure and could explain the large recovery of “lipids and lipid-like molecules” with their specific functions as reaction to the stress factors.

Taken together, we found very specific patterns of secondary metabolites in root exudates of the four cover crops (**Figures 3A**, **4**; **Table S1**). The bulk of recovered metabolites was cultivation-specific in every species (**Figure 2**), which implies that hydroponically-sampled root exudates poorly reflect the metabolic complexity of root exudates derived from the field. Especially, the presence of “lipids and lipid-like molecules” (containing also terpenoids) was highly indicative for field samples in all species (**Figures 3A**, **4**). However, gross metabolite *superclass*es are comparable to a large extent between hydro and field conditions (**Figure 4**). We identified a large amount of “phenylpropanoids and polyketides” as indicative for clover, while “organoheterocyclic compounds” or “benzenoids” were highly characteristic for mustard or oat irrespective of the cultivation condition. Also at the compound level, several compounds were highly abundant in both, hydroponically- and field-sampled exudates (**Table S1**). Quite prominent is the pattern of biochanin A, formononetin, putative trifolirhizin-6’-O-malonate, an unknown compound (m/z: 447.1281, RT: 6.49), kaempferol and putative soyasaponin I in clover. These compounds were on ranks 1-10 in either cultivation condition and were not present in any of the other species. Likewise, 6 species-specific compounds in mustard and 5 in oat were at least within the top 20 ranks and thus typical for these species (**Table S1**; Note: The 7^th^ compound in the top 20 of mustard with m/z=311.2187 and RT=6.97 was also present in oat hydro.). Based on these observations, we propose that the presence of distinct metabolites can serve as “finger prints” for individual species, and thereby help identifying the origin of soil solution samples in ecological studies, where the genetic and omics-based analysis of bulk samples from the environment is of rising importance.

Based on our results we conclude that sampling root exudates in hydroponic systems is a valid approach to identify the broad *superclasses* of metabolites released by different plant species. Field sampling approaches should be considered when investigating the exudation of specific compounds in response to environmental conditions. Then, field sampling approaches with low risk of root injury should be preferred.

## Author’s note

This manuscript is dedicated to Prof. Dr. Dierck Scheel, IPB Halle, Germany, who deceased in May 2022.

## Data availability statement

The original contributions presented in the study are included in the article/**Supplementary Material**. Further inquiries can be directed to the corresponding author (http://dx.doi.org/10.5447/ipk/2022/19).

## Author contributions

DH and NW conceived the study. DH and SD designed methodology. DH, SD, DS, UF and NG collected the data. DH and SD analyzed the data. DH and NW wrote the manuscript with contribution and approval of all authors. All authors contributed to the article and approved the submitted version.
