# Supplementary material for: Distinct metabolite classes in root exudates are indicative for field- or hydroponically-grown cover crops
Source: Front Plant Sci. 2023 Apr 6;14:1122285. doi: 10.3389/fpls.2023.1122285 (PMC10118039; doi:10.3389/fpls.2023.1122285)

**S1 Figure: Staining of roots of mustard, phacelia, oat and clover with trypan blue directly after removing them from field soil by manual washing.** Roots were stained directly after the washing procedure (0 h) and after two hours of root exudate sampling in darkened plastic bottles containing 1 L of deionized water with 5 mg L^-1^ Micropure forte (Katadyn, Mörfelden-Walldorf, Germany; 2 h). Roots were incubated in 0.4 % trypan blue solution for 1 min and washed with deionized water. Scale bars represent 250 µm.


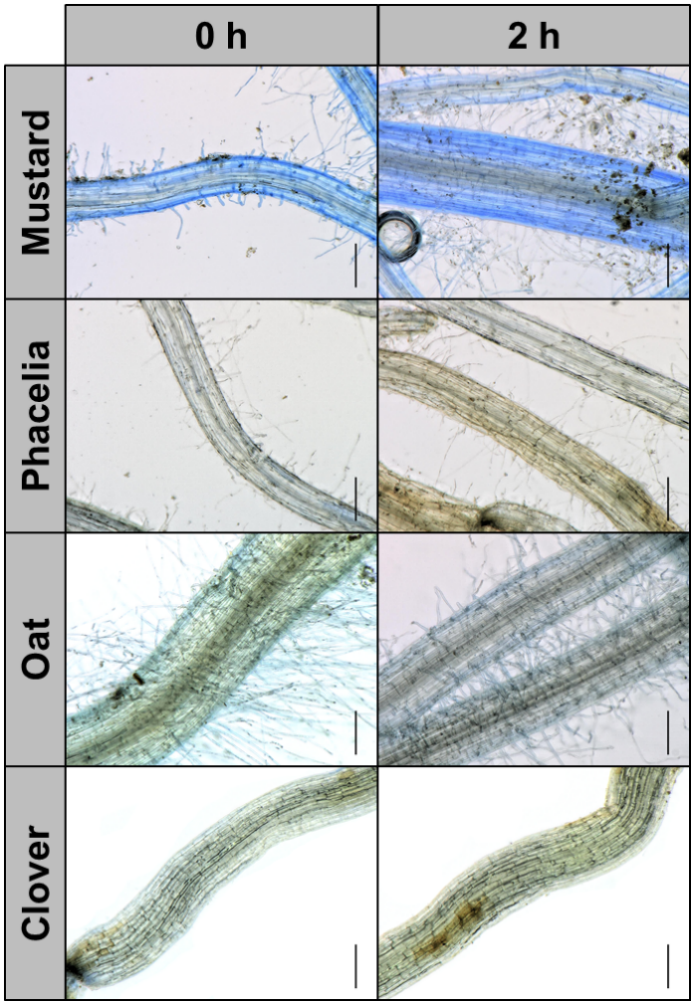

Supplement: Supplementary file 1 [file DataSheet_1.docx]
