# Supplementary material for: Distinct metabolite classes in root exudates are indicative for field- or hydroponically-grown cover crops
Source: Front Plant Sci. 2023 Apr 6;14:1122285. doi: 10.3389/fpls.2023.1122285 (PMC10118039; doi:10.3389/fpls.2023.1122285)
Supplement: Supplementary file 2 [file Table_1.docx]

**S1 Table: Putative chemical classification of the 100 most abundant features in root exudates of mustard, phacelia, oat and clover grown in the field (FIELD) or hydroponically (HYDRO), respectively.** Features were sorted by mean peak height and their rank among the top 100 in either growth condition are shown, n=9-11 [FIELD], n=3-4 [HYDRO]. Putative metabolites were annotated at three levels of confidence: Annotation level 1 indicates a match of m/z value, retention time (RT) and collision-induced dissociation spectrum, if available, to a commercial or synthetized standard; level 2 annotation has a good evidence according to matches of the mass spectrum with literature data, but was not verified by a standard; level 3 indicates automated annotation by MetaboScape software (Bruker Daltonics, Bremen, Germany). Metabolites were tentatively chemically classified using ClassyFire tool (http://classyfire.wishartlab.com; Djoumbou Feunang et al. (2016). Here, the *superclass* level is shown.

**S1 Table continued on next page. ►**

**S1 Table: Continued from previous page.**

**S1 Table continued on next page. ►**

**S1 Table: Continued from previous page.**

**S1 Table continued on next page. ►**

**S1 Table: Continued from previous page.**

**S1 Table continued on next page. ►**

**S1 Table: Continued from previous page.**

**S1 Table continued on next page. ►**

**S1 Table: Continued from previous page.**

**S1 Table continued on next page. ►**

**S1 Table: Continued from previous page.**

**S1 Table continued on next page. ►**

**S1 Table: Continued from previous page.**

**S1 Table continued on next page. ►**

**S1 Table: Continued from previous page.**

**S1 Table continued on next page. ►**

**S1 Table: Continued from previous page.**

**S1 Table continued on next page. ►**

**S1 Table: Continued from previous page.**

**S1 Table continued on next page. ►**

**S1 Table: Continued from previous page.**

**S1 Table continued on next page. ►**

**S1 Table: Continued from previous page.**

**S1 Table continued on next page. ►**

**S1 Table: Continued from previous page.**

**S1 Table continued on next page. ►**

**S1 Table: Continued from previous page.**

**S1 Table continued on next page. ►**

**S1 Table: Continued from previous page.**

**S1 Table continued on next page. ►**

**S1 Table: Continued from previous page.**

**S1 Table continued on next page. ►**

**S1 Table: Continued from previous page.**

**References**

Djoumbou Feunang, Y., Eisner, R., Knox, C., Chepelev, L., Hastings, J., Owen, G., Fahy, E., Steinbeck, C., Subramanian, S., Bolton, E., Greiner, R., Wishart, D.S., 2016. ClassyFire: automated chemical classification with a comprehensive, computable taxonomy. Journal of Cheminformatics 8, 61.
