# Supplementary material for: Distinct metabolite classes in root exudates are indicative for field- or hydroponically-grown cover crops
Source: Front Plant Sci. 2023 Apr 6;14:1122285. doi: 10.3389/fpls.2023.1122285 (PMC10118039; doi:10.3389/fpls.2023.1122285)
Supplement: Supplementary file 3 [file Table_2.docx]

**S2 Table: Fresh and dry weight of cover crop plant material in the field (FIELD) and in hydroponic culture (HYDRO).** Values show means ±SD (n=9-11 [FIELD], n=18-21 [HYDRO]); n.d. = not determined.

|  | **Mustard** | | **Phacelia** | | **Oat** | | **Clover** | |
| --- | --- | --- | --- | --- | --- | --- | --- | --- |
|  | **FIELD** | **HYDRO** | **FIELD** | **HYDRO** | **FIELD** | **HYDRO** | **FIELD** | **HYDRO** |
| **Organ weight [g plant^-1^]** | |  |  |  |  |  |  |  |
| *Fresh weight* |  |  |  |  |  |  |  |  |
| Shoot | n.d. | 18.3 ±7.6 | n.d. | 9.2 ±3.7 | n.d. | 19.9 ±2.5 | n.d. | 4.6 ±2.5 |
| Root | 1.7 ±0.6 | 2.6 ±1.3 | 0.7 ±0.4 | 4.3 ±1.6 | 1.5 ±0.4 | 9.3 ±1.9 | 0.9 ±0.3 | 1.9 ±30.9 |
| *Dry weight* |  |  |  |  |  |  |  |  |
| Shoot | 0.6 ±0.2 | 1.9 ±0.8 | 0.2 ±0.1 | 0.7 ±0.3 | 0.6 ±0.3 | 2.2 ±0.3 | 0.3 ±0.1 | 0.5 ±0.3 |
| Root | 0.3 ±0.04 | 0.2 ±0.1 | 0.07 ±0.04 | 0.14 ±0.04 | 0.2 ±0.1 | 0.5 ±0.1 | 0.09 ±0.05 | 0.11 ±0.04 |
| **Root:shoot dry weight ratio** | 0.48 ±0.12 | 0.10 ±0.03 | 0.35 ±0.09 | 0.19 ±0.07 | 0.33 ±0.11 | 0.22 ±0.02 | 0.29 ±0.09 | 0.23 ±0.05 |
